# Growth hormone treatment improves final height in children with X-linked hypophosphatemia

**DOI:** 10.1186/s13023-022-02590-5

**Published:** 2022-12-21

**Authors:** Julia André, Volha V. Zhukouskaya, Anne-Sophie Lambert, Jean-Pierre Salles, Brigitte Mignot, Claire Bardet, Catherine Chaussain, Anya Rothenbuhler, Agnès Linglart

**Affiliations:** 1 AP-HP, Endocrinology and Diabetes for Children, Reference Center for Rare Diseases of Calcium and Phosphate Metabolism, DMU SEA, OSCAR Filière, EndoRare and BOND ERN, Bicêtre Paris Saclay Hospital, 78 Rue du Général Leclerc, 94270 Le Kremlin Bicêtre, France; 2 Laboratory Orofacial Pathologies, Imaging and Biotherapies URP2496 and FHU-DDS-Net, Dental School, and Plateforme d’Imagerie du Vivant (PIV), Université Paris Cité, Montrouge, France; 3 AP-HP, Medicine for Adolescents, Bicêtre Paris Saclay Hospital, Le Kremlin Bicêtre, France; 4 Unit of Endocrinology and Bone Diseases, Children Hospital, Toulouse University Hospital, CHU de Toulouse, Université de Toulouse, ERN BOND, INSERM UMR 1291/CNRS 5051, INFINITY Center, Toulouse, France; 5 Department of Pediatrics, CHU of Besancon, Besançon, France; 6 AP-HP, Reference Center for Rare Disorders of the Calcium and Phosphate Metabolism, Dental Medicine Department, Bretonneau Hospital, GHN-Universite de Paris, Paris, France; 7 INSERM, Physiologie Et Physiopathologie Endocrinienne, Bicêtre Paris Saclay Hospital, Paris Saclay University, Le Kremlin Bicêtre, France

**Keywords:** X-linked hypophosphatemia, Rickets, Fibroblast growth factor 23, Recombinant human growth hormone, Final height

## Abstract

**Background/aim:**

Despite optimal conventional treatment (oral phosphate supplements and active vitamin D analogs), about 40–50% of children with well-controlled X-linked hypophosphatemia (XLH) show linear growth failure, making them less likely to achieve an acceptable final height. Here, we studied the hypothesis that rhGH treatment improves final height in children with XLH and growth failure.

**Methods:**

Two cohorts of children with XLH were included in this retrospective longitudinal analysis: (1) a cohort treated with rhGH for short stature (n = 34) and (2) a cohort not treated with rhGH (n = 29). The mean duration of rhGH treatment was 4.4 ± 2.9 years. We collected the auxological parameters at various time points during follow-up until final height.

**Results:**

In rhGH-treated children, 2 years of rhGH therapy was associated with a significant increase in height from − 2.4 ± 0.9 to − 1.5 ± 0.7 SDS (*p* < 0.001). Their mean height at rhGH discontinuation was − 1.2 ± 0.9 SDS and at final height was − 1.3 ± 0.9 SDS corresponding to 165.5 ± 6.4 cm in boys and 155.5 ± 6.3 cm in girls. Notably, the two groups had similar final heights; i.e., the final height in children not treated with rhGH being − 1.2 ± 1.1 SDS (165.4 ± 6.8 cm in boys and 153.7 ± 7.8 cm in girls), *p* = 0.7.

**Conclusion:**

Treatment with rhGH permits to improve final height in children with XLH and growth failure, despite optimal conventional treatment. We propose therefore that rhGH therapy could be considered as an option for short stature in the context of XLH.

**Supplementary Information:**

The online version contains supplementary material available at 10.1186/s13023-022-02590-5.

## Introduction

X-linked hypophosphatemia (XLH) is the most common form of hereditary rickets with an incidence of 3.9–5 per 100,000 live births [1]. It is a genetic disease caused by loss of function mutations in the phosphate-regulating gene with homologies to endopeptidases on the X chromosome (*PHEX*) gene, localized on Xp22.1. *PHEX* encodes an endopeptidase highly expressed in bone (osteoblasts, osteocytes) and tooth (odontoblasts) cells [2]. Animal studies indicate that loss of *PHEX* function results in enhanced secretion of the phosphaturic hormone fibroblast growth factor 23 (FGF23), which leads to renal phosphate wasting, hypophosphatemia and insufficient calcitriol (1,25(OH)_2_D) synthesis [3, 4]. Chronic hypophosphatemia and low levels of 1,25(OH)_2_D lead to mineralization failure which, in children with XLH, manifests clinically as rickets, e.g., severe skeletal deformities and short stature, recurrent dental abscesses and craniosynostosis. Poor growth and disproportionate short stature are often the first symptoms of XLH in children. In children with XLH, length at birth is reported as normal [1] but growth retardation becomes notable during the first years of life [5, 6], resulting in a mean height in these patients 2 standard deviation scores (SDS) below the mean height of the reference population [7, 8]. Despite adequate conventional treatment (phosphate supplementations and active vitamin D analogs), even when started early in life, the growth retardation may persist. Indeed, most studies have reported that about half of treated patients still have short stature at the end of growth (< − 2 SDS) [9–11] which results in reduced final adult height [5, 7, 9, 12–14].

## Background

Treatment with recombinant human growth hormone (rhGH) has been recently proposed as a support to improve linear growth in children with XLH showing growth failure regardless of well controlled disease. It is known that GH exerts its effect on growth cartilage through the action of insulin-like growth factor-1 (IGF-1), which plays an important role in the differentiation and maturation of chondrocytes of the growth plate. IGF-1 also stimulates osteoblastogenesis and enhances collagen secretion and bone matrix mineralization. In osteocytes, IGF-1 helps in transducing mechanical stimuli and promoting the anabolic response. Additionally, the IGF system interacts with other hormones, such as parathyroid hormone and sex steroids, promoting bone anabolism [15]. Moreover, it is thought that GH, directly and indirectly through IGF-1, may increase renal phosphate reabsorption and thereby serum phosphate levels [16–19]. Indeed, receptors for GH and IGF-I have been identified in the proximal renal tubule. In line with this, it has been shown that the positive effect of rhGH administration on phosphate metabolism is likely related to the stimulation of renal phosphate reabsorption and 1α-hydroxylase activity [20–22]. It is interesting to note that patients with XLH may have a transient increase in serum phosphate during rhGH treatment attributable to a transient decrease in urinary phosphate excretion [16]. In support of these facts, several clinical studies have demonstrated beneficial effect of rhGH on growth, especially if started at a prepubertal age [16, 17, 20–24], and importantly, no side effects have been reported during rhGH treatment. On the other hand, only one study [25] has explored whether the height is maintained after rhGH withdrawal, and especially, whether rhGH treatment improves final height in XLH.

Proposal of the current study is to demonstrate the effect of rhGH on final height in children with XLH and poor growth.

## Results

### The whole cohort of children with XLH

The characteristics of the cohort included in the present study are summarized in Table 1. We selected 63 children with XLH (23 boys and 40 girls) among which 56 (89%) carried the *PHEX* mutation. Thirty-four of these children received treatment with rhGH for persistent short stature despite adequate conventional treatment; the other 29 were not given rhGH because of adequate growth or because they decided not to take this additional therapy and to continue conventional treatment. Comparing the two groups, we did not find any differences in the distribution of boys and girls, the rate of *PHEX* mutations, age at XLH diagnosis or duration of conventional treatment. Interestingly, rhGH-treated children tended to have lower birth weight and length, than those not treated with rhGH, but this difference did not reach statistical significance.

**Table 1:** Characteristics of children with X-liked hypophosphatemia included in the study

Parameters	Patients treated with rhGH	Patients not treated with rhGH	*p*
	Mean ± SD [min–max] or n (%)	Mean ± SD [min–max] or n (%)	
Number of patients	34	29	–
Boys/girls, n (%)	13 (38%)/21 (62%)	10 (34%)/19 (66%)	0.49
Birth weight, SDS	− 0.5 ± 2.0	0.4 ± 1.2	0.11
	[− 5.0 to 3.5]	[− 1.5 to 2.0]	
Birth length, SDS	− 0.5 ± 1.9	0.5 ± 1.2	0.09
	[− 5.0 to 3.0]	[− 1.7 to 1.9]	
Patients carrying a *PHEX* mutation, n (%)	32 (94%)	24 (86%)	0.29
Age at XLH diagnosis, years	3.4 ± 3.4	2.6 ± 2.6	0.32
	[0.1–12]	[0.0–11.5]	
Duration of conventional therapy, years	12.6 ± 4.9	14.0 ± 3.0	0.20
	[3.0–21.8]	[5.5–17.8]	
Age at menarche, years	13.4 ± 1.3	12.8 ± 0.8	0.15
	[9.8–15.1]	[11.3–14.4]	
Age at rhGH start, years	9.8 ± 3.4	–	–
	[2.5–15.2]		
Duration of rhGH treatment, years	4.4 ± 2.9	–	–
	[0.6–12]		
Dose of rhGH (µg/kg/day)	77.4 ± 14.5	–	–
– at the beginning of treatment	77.4 ± 14.5		
– at the end of treatment	66.8 ± 20.5		
Patients treated with a GnRH, n (%)	15 (47%)	2 (7%)	< 0.000

rhGH: Recombinant human growth hormone; aGnRH: gonadotropin-releasing hormone analog; SDS: standard deviation score or Z-score

### Final height under rhGH treatment

**Fig. 1:**
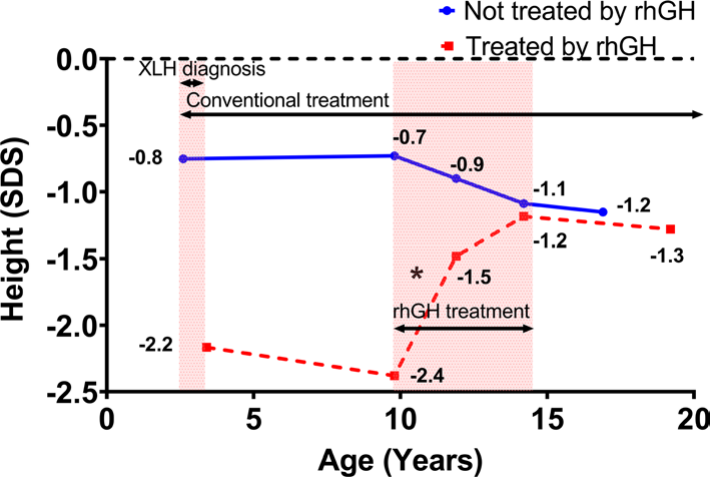
Height SDS in children with X-linked hypophosphatemia from diagnosis to adult height. Changes in height of children with XLH who received rhGH are plotted with the red dotted line. Children treated with rhGH were diagnosed with XLH at a mean age of 3.4 years. The rhGH treatment was started at average of 9.8 years and discontinued at average of 14.2 years. The age at last visit was ~ 19.2 years. Changes in height of children with XLH who did not receive rhGH are plotted with the blue line. The children in this cohort were diagnosed with XLH at a mean age of 2.6 years. The age at last visit was at average of 16.9 years. *p < 0.001. XLH: X-linked hypophosphatemia; SDS: standard deviation score or Z-score; rhGH: recombinant human growth hormone

Thirty-four patients treated with rhGH started treatment at a mean age of 9.8 ± 3.5 years and continued it for 4.4 ± 2.9 years. The changes in height SDS from diagnosis to final height are shown in Table 2 and Fig. 1.

**Table 2:** Changes in height SDS of children with X-linked hypophosphatemia followed-up until final height

Time points of follow-up	Patients treated with rhGH (n = 34)		Patients non treated with rhGH (n = 29)	
	Age, years	Height SDS	Age, years	Height SDS
	Mean ± SD	Mean ± SD	Mean ± SD	Mean ± SD
Birth	–	− 0.5 ± 1.9	–	0.5 ± 1.2
Diagnosis	3.5 ± 3.4	− 2.2 ± 1.2	2.6 ± 2.6	− 0.8 ± 1.6
Start of rhGH	9.8 ± 3.5	− 2.4 ± 0.9	9.8 ± 0.0	− 0.7 ± 0.9
After 2 years of rhGH	11.8 ± 3.4	− 1.4 ± 0.7	11.9 ± 0.0	− 0.9 ± 0.9
At the end of rhGH	14.2 ± 3.1	− 1.2 ± 0.9	14.2 ± 0.0	− 1.1 ± 1.1
Final height	18.3 ± 3.2	− 1.3 ± 0.9	16.9 ± 2.1	− 1.2 ± 1.1
		165.5 ± 6.4 cm in boys 155.5 ± 6.3 cm in girls		165.4 ± 6.8 cm in boys 153.7 ± 7.8 cm in girls
Height gain at the end of rhGH	1.2 ± 0.7		–	–
Height gain at final height	1.2 ± 0.9		–	–

rhGH: Recombinant human growth hormone; SDS: standard deviation score or Z-score

As expected [10], rhGH-treated children had significantly shorter stature at XLH diagnosis (− 2.2 ± 1.2 SDS) than those who did not receive rhGH (− 0.8 ± 1.6 SDS, *p* < 0.001). In the former group of children height did not improve, remaining at − 2.4 ± 0.9 SDS after almost 7 years of conventional treatment. Having started rhGH at a mean age of 9.8 ± 3.5 years, their height SDS was significantly higher than baseline after 2 years of treatment, at the end of rhGH treatment and at final height (− 1.5 ± 0.7, *p* = 0.003, − 1.2 ± 0.9, *p* < 0.001 and − 1.3 ± 0.9 SDS, *p* < 0.001 respectively). Notably, the height SDS gain was maintained until final height despite the discontinuation of rhGH. The total height gain was 1.2 ± 0.9 SDS. Out of these 34 children treated with rhGH, 75% reached a final height >  − 2 SDS. Importantly, the final height in children of this cohort was not significantly different from children in the cohort of children who did not receive rhGH (− 1.3 ± 0.9 vs. − 1.2 ± 1.1 SDS, *p* = 0.7).

Interestingly, the rate of rhGH treatment was somewhat higher in children with a de novo diagnosis of XLH (without a family history of XLH) than those with a family history, though the difference did not reach significance (64.3% vs. 43.3%, *p* = 0.091).

### Subgroup analysis of children treated with rhGH + aGnRH

As mentioned in the methods, a small group of children (n = 17; 27%) with clinical signs of early puberty was treated with GnRH analog (aGnRH); 15 (47%) of the 34 rhGH-treated children had also received aGnRH in addition to rhGH, a rate higher than among those were not given rhGH (n = 2; 7%) (*p* < 0.001). To explore potential bias, we performed a subgroup analysis in the group of children treated with rhGH and aGnRH (Additional file 1: Tables S1 and S2). No differences were detected between the subgroups (treated with rhGH + aGnRH and only with rhGH) in terms of the number of patients carrying the *PHEX* mutation, gender distribution, gestational age/weight/length at birth, or duration of conventional and rhGH treatment (Additional file 1: Table S1). The height SDS did not differ significantly between the two subgroups (treated and not treated with aGnRH) at any points during follow-up (Additional file 1: Table S2).

## Discussion

In this retrospective longitudinal observational study, we show for the first time that treatment with rhGH improves final height in children with XLH who manifest short stature despite well controlled disease.

Several studies have demonstrated beneficial effects of rhGH on growth in XLH [16, 17, 20–24], but few data are available regarding its effect on final height. Exceptions include data from the study by Baroncelli et al. [21] on a small group of children with XLH (n = 6) treated with rhGH suggesting it has a positive effect on adult height. Further, we recently conducted a study that showed that 2 years of treatment with rhGH is associated with a significant improvement in height and growth velocity in children with XLH [24]. This present study is the first to describe and consolidate the notion that rhGH has a positive effect on final height in XLH children with short stature. Indeed, we demonstrate that the treatment with rhGH is associated with an increase in final height in children who still manifested short stature despite optimal conventional treatment. The height gain obtained was most significant during the first 2 years of treatment and the gain was sustained until the achievement of final height, despite the rhGH discontinuation. Most importantly, treatment with rhGH allows these children to achieve an acceptable final height comparable to that of children who did not receive rhGH (165.5 ± 6.4 cm in boys and 155.5 ± 6.3 cm in girls). At final height, of the whole cohort of 63 patients, 79.5% reached a height >  − 2 SDS (75% in the cohort treated with rhGH and 83.3% in the cohort non treated with rhGH).

The effect of XLH on growth is independent of disorders of growth hormone (GH) secretion/action or nutritional deficiencies [26], and only few patients with XLH have a GH deficit [5, 27]. Impaired bone elongation in XLH resides at least partly in structural abnormalities of the growth plate, as it was described in a murine model of XLH (*Hyp* mice) by Fuente et al., which hinder normal endochondral ossification [28]. These abnormalities are, first of all, due to hypophosphatemia which attenuates chondrocyte differentiation and hypertrophic chondrocyte apoptosis leading to an increase in the hypertrophic layer of the growth plate [29]. Secondly, FGF23 inhibits chondrocyte differentiation and might also have a causative role in growth retardation in XLH [30, 31]. The latter could probably explain the failure to achieve acceptable growth using conventional treatment with phosphate supplementation and active vitamin D analogs.

Currently, there are no widely accepted guidelines for the prescribing of rhGH to treat short stature in XLH children. Nonetheless, the recent clinical recommendations on the diagnosis and management of XLH [4] state that rhGH may be considered for children with XLH who present short stature despite well-controlled disease (normal levels of alkaline phosphatase and parathyroid hormone). In our study, the indications for rhGH treatment were height ≤  − 2 SDS despite well-controlled rickets on conventional treatment at least for 1 year and a height prognosis ≤  − 2.5 SDS.

Another open question regarding rhGH treatment is how long it should be given. In the majority of studies, the duration of rhGH treatment varies from 1 to 6 years [16, 19, 22, 27, 32–34]. Nonetheless, we observed that the acceleration of growth takes place during the first 2 years of treatment without any further significant improvement in height afterwards. Following the discontinuation of rhGH, height SDS remained stable until final height was reached. Therefore, it could be hypothesized that the duration of rhGH treatment could be limited to 2 years, given the constraints of the treatment (daily subcutaneous injections). On the other hand, it has been well demonstrated that it is important to give rhGH treatment throughout the whole period of accelerated growth to maintain the benefit of this treatment on height through the direct action on the growth plate [35].

The doses of rhGH treatment in our study were relatively high, averaging 77 µg/kg/day at initiation and 67 µg/kg/day at discontinuation. This contrasts with other previous research in which the average dose of rhGH was about 30 µg/kg/day, though these researchers failed to show a beneficial impact of rhGH on growth acceleration [36]. This suggests that higher doses of rhGH may be needed to promote growth in XLH. If such treatment is given, rhGH doses should be adjusted every 3–4 months to keep IGF-1 blood levels below 2 SDS [37, 38].

Finally, almost half of children treated with rhGH had also been treated with aGnRH, a treatment blocking puberty, thus influencing final height. We found no significant differences in final height between the subgroups of children who were and were not treated with aGnRH in addition to rhGH. By performing this subgroup analysis, we ruled out potential bias from the possibility that the positive effect of rhGH treatment on final height could depend on aGnRH treatment.

We recognize that the present study is retrospective with all the limitations that this entails. On the other hand, its strengths include the longitudinal design and the fact that it was performed in a large cohort of children with a disease as rare as XLH.

In conclusion, we believe that, to achieve an acceptable final height, rhGH may be an appropriate option for children with XLH who still have short stature despite well-controlled disease. We would thus recommend implementing rhGH treatment in the therapeutic management of XLH for children who do not grow correctly. Though the availability of novel therapeutic strategies for XLH, namely anti-FGF23 antibodies, has recently changed XLH landscape, the results of this study may help prescribing a more individualized treatment as the disease manifestations are very variable between patients even in affected siblings.

## Material and methods

### Patients

The present retrospective longitudinal observational study was conducted in the Reference Center for Rare Disorders of Calcium and Phosphate Metabolism, Filière OSCAR and Platform of expertise for rare diseases Paris Saclay, Bicêtre Paris Saclay Hospital, France. The diagnosis of XLH was based on clinical and/or radiological evidence of rickets, biochemical evidence of phosphate wasting associated with elevated levels of FGF23 and/or a variant in the *PHEX* gene. As soon as a child was diagnosed with XLH, we proceeded with a clinical examination of parents which involves a detailed clinical history focused on possible XLH manifestations during childhood such as leg deformities, recurrent dental abscesses, poor growth, short stature, and pain; measurement of serum phosphate levels; and genetic analysis for the mutations in *PHEX*, *DMP1, FGF23, ENPP1, FAM20C, FGFR1, KLOTHO* genes. In the absence of clinical and biochemical signs of hypophosphatemia, a subject was considered as non-affected with XLH. After diagnosis, all children started treatment with active vitamin D analogs (alfacalcidol) and phosphate supplements according to international recommendations [4].

In the present study, we included children with XLH who (i) were born between 1988 and 2006; (ii) regularly visited in our hospital, and therefore, had a detailed growth chart available; and (iii) had reached their final height. The whole cohort included children with XLH who were treated with rhGH and also those who did not need rhGH treatment (serving as “control group”). Children who underwent lower limb surgery were excluded from the analysis.

The study was approved by the French National Data Processing and Liberties Commission (CNIL number 1920371v0), and in accordance with the Jardé law in France, the need for written consent was waived. After patients and/or their parents had been informed orally about the study, oral consent was obtained from parents and oral assent from minors (as appropriate). It was clarified that they had the right to withdraw from the study at any time by completing a form available through http://recherche.aphp.fr/eds/droit-opposition.

### Treatments

Treatment with rhGH was given to children who (i) had a height ≤  − 2 SDS, ***(***ii) had received at least 1 year of adequate conventional therapy, and ***(***iii) had a height prognosis ≤  − 2.5 SDS. A thorough investigation was performed to exclude other causes of short stature. Some patients treated with rhGH were included in the prospective study promoted by INSERM and have been reported in Rothenbuhler and al. [24]. In all cases, rhGH was given as a subcutaneous injection 6 days a week; mean doses of rhGH at the beginning and withdrawal were 77.4 ± 14.5 and 66.8 ± 20.5 µg/kg/day, respectively. All children were compliant to the rhGH treatment.

A small group of children in both groups exposed or not to rhGH treatment who presented clinical signs of early puberty received aGnRH (leuprorelin, sustained-release formulation at a dose of 11.25 mg every 3 months) to block early puberty. The decision to add the aGnRH treatment was normally made by the pediatric endocrinology team after careful evaluation. Patients and their families were informed and gave their consent to this therapy.

### Study protocol and methods

The follow-up period was from disease diagnosis until the last visit. For each patient, we gathered data on parameters such as gestational age at birth, weight, length/height and body mass index (BMI) at several time points. For children treated with rhGH, we retrieved data at birth, at the time of XLH diagnosis, at the start of rhGH treatment, after 2 years of rhGH treatment, at the end of rhGH treatment and at the last visit, i.e., at final height. For children who did not receive rhGH treatment (the “control”), we collected the same types of data at equivalent stages during follow-up, namely, at birth, at the time of XLH diagnosis, at the ages at which patients in the other group initiated rhGH, 2 years later and at which they discontinued the treatment (i.e., mean ages of 9.8, 11.9 and 14.2 years, respectively) and at the last visit.

BMI was calculated using the formula weight (kg)/height (m)^2^. Z-scores (SDS) for length/height, weight, and BMI were derived from the World Health Organization growth charts [39]. Z-scores for weight were calculated only for children between 0 and 10 years of age. Final height was defined as height achieved at the completion of growth, i.e., when bone age > 16 years and/or growth velocity < 2 cm/year [40, 41].

### Statistical analysis

Statistical analysis was performed using IBM SPSS (Statistical Package for the Social Sciences) Statistics for Windows, version 25.0 (IBM Corp., Armonk, NY, USA). The results were expressed as mean ± SD for continuous variables and as absolute number (percentage) for categorical variables. The comparisons of continuous variables were performed using Student’s *t* test or the Mann–Whitney U test or using one-way analysis of variance, as appropriate. All *p* values < 0.05 were considered significant. Figures were plotted using the Prism Graph Pad software version 8.4.1.

## Supplementary Information

**Additional file 1: Table S1** Description of children with X-linked hypophosphatemia treated with rhGH and with or without aGnRH. **Table S2** Description of height changes in children with X-linked hypophosphatemia given rhGH through follow-up, stratified by whether they received aGnRH.

## Data Availability

The datasets generated and analysed during the current study are not publicly available but are available from the corresponding author on reasonable request.

## Abbreviations

XLH: X–linked hypophosphatemia
PHEX: Phosphate-regulating gene with homologies to endopeptidases on the X chromosome
FGF23: Fibroblast growth factor 23
SDS: Standard deviation score
rhGH: Recombinant human growth hormone
aGnRH: Analog of gonadotropin releasing hormone

## References

[1] Ruppe MD. X-linked hypophosphatemia. Gene Rev. 2017; 22.

[2] Francis F (1995). A gene (PEX) with homologies to endopeptidases is mutated in patients with X-linked hypophosphatemic rickets. Nat Genet.

[3] Liu S, Tang W, Zhou J, Stubbs JR, Luo Q, Pi M (2006). Fibroblast growth factor 23 is a counter-regulatory phosphaturic hormone for vitamin D. JASN.

[4] Haffner D, Emma F, Eastwood DM, Duplan MB, Bacchetta J, Schnabel D (2019). Clinical practice recommendations for the diagnosis and management of X-linked hypophosphataemia. Nat Rev Nephrol.

[5] Mäkitie O, Doria A, Kooh SW, Cole WG, Daneman A, Sochett E (2003). Early treatment improves growth and biochemical and radiographic outcome in X-linked hypophosphatemic rickets. J Clin Endocrinol Metab.

[6] Cagnoli M (2017). Spontaneous growth and effect of early therapy with calcitriol and phosphate in X-linked hypophosphatemic rickets. Pediatr Endocrinol Rev.

[7] Steendijk R, Hauspie RC (1992). The pattern of growth and growth retardation of patients with hypophosphataemic vitamin D-resistant rickets: a longitudinal study. Eur J Pediatr.

[8] GunnarB S, BruceZ M (1989). Hypophosphatemic rickets: final height and clinical symptoms in adults. Lancet.

[9] Haffner D, Weinfurth A, Manz F, Schmidt H, Bremer HJ, Mehls O (1999). Long-term outcome of paediatric patients with hereditary tubular disorders. Nephron.

[10] Linglart A, Biosse-Duplan M, Briot K, Chaussain C, Esterle L, Guillaume-Czitrom S (2014). Therapeutic management of hypophosphatemic rickets from infancy to adulthood. Endocr Connect.

[11] Jehan F, Gaucher C, Nguyen TM, Walrant-Debray O, Lahlou N, Sinding C (2008). Vitamin D receptor genotype in hypophosphatemic rickets as a predictor of growth and response to treatment. J Clin Endocrinol Metab.

[12] Balsan S, Tieder M (1990). Linear growth in patients with hypophosphatemic vitamin D-resistant rickets: Influence of treatment regimen and parental height. J Pediatr.

[13] Reusz GS. Hypophosphataemic rickets. The Lancet. 1990; 178 10.1016/0140-6736(90)90058-d 1967476

[14] Berndt M (1996). Clinical course of hypophosphatemic rickets in 23 adults. Clin Nephrol.

[15] Yakar S. Insulin-like growth factors: actions 1 on the skeleton. J Mol Endocrinol 2018; 17‑0298. 10.1530/JME-17-0298 PMC5966339 29626053

[16] Seikaly MG, Brown R, Baum M (1997). The effect of recombinant human growth hormone in children with X-linked hypophosphatemia. Pediatrics.

[17] Živičnjak M, Schnabel D, Staude H, Even G, Marx M, Beetz R (2011). Three-year growth hormone treatment in short children with X-linked hypophosphatemic rickets: effects on linear growth and body disproportion. J Clin Endocrinol Metab.

[18] On Behalf of the Hypophosphatemic Rickets Study Group of the “Deutsche Gesellschaft für Kinderendokrinologie und -diabetologie” and “Gesellschaft für Pädiatrische Nephrologie”, Meyerhoff N, Haffner D, Staude H, Wühl E, Marx M, et al. Effects of growth hormone treatment on adult height in severely short children with X-linked hypophosphatemic rickets. Pediatr Nephrol. 2018;33(3):447‑56. 10.1007/s00467-017-3820-3 29058153

[19] Reusz GS, Miltényi G, Stubnya G, Szabó A, Horváth C, Byrd DJ (1997). X-linked hypophosphatemia: effects of treatment with recombinant human growth hormone. Pediatr Nephrol.

[20] Saggese G, Baroncelli GI, Bertelloni S, Perri G (1995). Long-term growth hormone treatment in children with renal hypophosphatemic rickets: effects on growth, mineral metabolism, and bone density. J Pediatr.

[21] Hammerman MR. The growth hormone-insulin-like growth factor axis in kidney. 12

[22] Mäkitie O, Toiviainen-Salo S, Marttinen E, Kaitila I, Sochett E, Sipilä I (2008). Metabolic control and growth during exclusive growth hormone treatment in X-linked hypophosphatemic rickets. Horm Res Paediatr.

[23] Rothenbühler A, Piquard C, Gueorguieva I, Lahlou N, Linglart A, Bougnères P (2010). Near normalization of adult height and body proportions by growth hormone in pycnodysostosis. J Clin Endocrinol Metab.

[24] Rothenbuhler A, Esterle L, Gueorguieva I, Salles JP, Mignot B, Colle M (2017). Two-year recombinant human growth hormone (rhGH) treatment is more effective in pre-pubertal compared to pubertal short children with X-linked hypophosphatemic rickets (XLHR). Growth Hormon IGF Res.

[25] Baroncelli GI, Bertelloni S, Ceccarelli C, Saggese G (2001). Effect of growth hormone treatment on final height, phosphate metabolism, and bone mineral density in children with X-linked hypophosphatemic rickets. J Pediatr.

[26] Fuente R, Gil-Peña H, Claramunt-Taberner D, Hernández O, Fernández-Iglesias A, Alonso-Durán L (2017). X-linked hypophosphatemia and growth. Rev Endocr Metab Disord.

[27] Schutt S, Schumacher M, Holterhus P, Felgenhauer S, Hiort O (2003). Effect of GH replacement therapy in two male siblings with combined X-linked hypophosphatemia and partial GH deficiency. Eur J Endocrinol.

[28] Fuente R, Gil-Peña H, Claramunt-Taberner D, Hernández-Frías O, Fernández-Iglesias Á, Hermida-Prado F (2018). Marked alterations in the structure, dynamics and maturation of growth plate likely explain growth retardation and bone deformities of young Hyp mice. Bone.

[29] Liu ES, Zalutskaya A, Chae BT, Zhu ED, Gori F, Demay MB (2014). Phosphate interacts with PTHrP to regulate endochondral bone formation. Endocrinology.

[30] Kawai M, Kinoshita S, Kimoto A, Hasegawa Y, Miyagawa K, Yamazaki M (2013). FGF23 suppresses chondrocyte proliferation in the presence of soluble α-Klotho both in vitro and in vivo. J Biol Chem.

[31] Raimann A, Ertl DA, Helmreich M, Sagmeister S, Egerbacher M, Haeusler G (2013). Fibroblast growth factor 23 and Klotho are present in the growth plate. Connect Tissue Res.

[32] Haffner D, Nissel R, Wuhl E, Mehls O (2004). Effects of growth hormone treatment on body proportions and final height among small children with X-linked hypophosphatemic rickets. Pediatrics.

[33] Yang HM, Mao M, Yang F, Wan C. Recombinant growth hormone therapy for X-linked hypophosphatemia in children. Cochrane Cystic Fibrosis and Genetic Disorders Group, éditeur. Cochrane Database of Systematic Reviews [Internet]. 24 janv 2005 [cité 31 mars 2020]; Disponible sur: 10.1002/14651858.CD004447.pub2

[34] Santos F, Fuente R, Mejia N, Mantecon L, Gil-Peña H, Ordoñez FA (2013). Hypophosphatemia and growth. Pediatr Nephrol.

[35] Labarta JI (2020). Growth and metabolic effects of long-term recombinant human growth hormone (rhGH) treatment in short children born small for gestational age: GH-RAST study. J Pediatr Endocrinol Metab..

[36] Cameron S, Daneman K (1999). A trial of growth hormone therapy in well-controlled hypophosphataemic rickets. Clin Endocrinol.

[37] Cohen P, Germak J, Rogol AD, Weng W, Kappelgaard AM, Rosenfeld RG (2010). Variable degree of growth hormone (GH) and insulin-like growth factor (IGF) sensitivity in children with idiopathic short stature compared with GH-deficient patients: evidence from an IGF-based dosing study of short children. J Clin Endocrinol Metab.

[38] Cohen P, Rogol AD, Howard CP, Bright GM, Kappelgaard AM, Rosenfeld RG (2007). Insulin growth factor-based dosing of growth hormone therapy in children: a randomized, controlled study. J Clin Endocrinol Metab.

[39] The WHO. The WHO child growth standards. Available at http://www.who.int/childgrowth/standards/en/.

[40] Ross JL, Lee PA, Gut R, Germak J (2015). Increased height standard deviation scores in response to growth hormone therapy to near-adult height in older children with delayed skeletal maturation: results from the ANSWER program. Int J Pediatr Endocrinol.

[41] Wu D, Chen R, Chen S, Liu G, Chen L, Yang Y (2020). Final adult height of children with idiopathic short stature: a multicenter study on GH therapy alone started during peri-puberty. BMC Pediatr.

