# Supplementary material for: Growth hormone treatment improves final height in children with X-linked hypophosphatemia
Source: Orphanet J Rare Dis. 2022 Dec 21;17:444. doi: 10.1186/s13023-022-02590-5 (PMC9768884; doi:10.1186/s13023-022-02590-5)
Supplement: Supplementary file 1 — Additional file 1: Table S1 Description of children with X-linked hypophosphatemia treated with rhGH and with or without aGnRH. Table S2 Description of height changes in children with X-linked hypophosphatemia given rhGH through follow-up, stratified by whether they received aGnRH. [file 13023_2022_2590_MOESM1_ESM.docx]

**Supplementary Table 1:** Description of children with X-linked hypophosphatemia treated with rhGH and with or without aGnRH.

| **Parameter** | **Patients treated with**  **rhGH + aGnRH**  **mean±SD [min-max] or**  **n (%)** | **Patients treated with**  **rhGH without aGnRH**  **mean±SD [min-max] or**  **n (%)** | **p** |
| --- | --- | --- | --- |
| Number of patients | 15 | 19 | - |
| Boys /  Girls, n (%) | 5 (33%) /  10 (67%) | 8 (42%) /  11 (58%) | - |
| Patients carrying *PHEX* mutation, n (%) | 14 (93%) | 15 (79%) | - |
| Birth term, weeks of amenorrhea | 39.2±2.07  [37.5 – 41.0] | 39.2±1.03  [34.0 – 42.0] | 0.97 |
| Birth weight, SDS | -0.5±2.1  [-5.0 – 2.2] | -0.6±2.2  [-5.0 – 3.5] | 0.91 |
| Birth length, SDS | -0.5±2.3  [-5.0 – 3.0] | -0.6±1.6  [-4.0 – 2.1] | 0.95 |
| Duration of conventional therapy, years | 13.7±4.5  [3.7-19] | 11.6±5.0  [3.0-21.8] | 0.20 |
| Duration of rhGH treatment, years | 5.1±2.8  [1.1-12] | 3.8±2.9  [0.6-10] | 0.19 |
| Age of menarche, years | 13.3±1.6  [9.8-15.1] | 13.6±1  [11.9-14.5] | 0.62 |

XLH: X-linked hypophosphatemia; rhGH: recombinant human growth hormone; GnRH: gonadotropin-releasing hormone; SDS: standard deviation score or Z-score

**Supplementary Table 2:** Description of height changes in children with X-linked hypophosphatemia given rhGH through follow-up, stratified by whether they received aGnRH

| **Time points of follow-up** | **Age, years** | **Height SDS,**  **mean±SD** | | **p** |
| --- | --- | --- | --- | --- |
|  |  | **Patients treated**  **with rhGH + aGnRH** | **Patients treated**  **with rhGH without aGnRH** |  |
| At XLH diagnosis | 3.4±3.4 | -2.3±1.6 | -2.1±0.7 | 0.7 |
| Before starting rhGH treatment | 9.8±3.5 | -2.2±1.0 | -2.5±0.7 | 0.3 |
| After 2 years of rhGH treatment | 11.9±3.4 | -1.6±0.8 | -1.4±0.6 | 0.6 |
| At the end of rhGH treatment | 14.2±3.1 | -1.2±0.9 | -1.1±0.8 | 0.7 |
| Final height | 19.2±3.4 | -1.2±0.9 | -1.3±1.0 | 0.8 |

XLH: X-linked hypophosphatemia; rhGH: recombinant human growth hormone; SDS: standard deviation score or Z-score; aGnRH: gonadotropin-releasing hormone agonis
